# Reduced mitochondrial mass and function add to age‐related susceptibility toward diet‐induced fatty liver in C57BL/6J mice

**DOI:** 10.14814/phy2.12988

**Published:** 2016-09-30

**Authors:** Kerstin Lohr, Fiona Pachl, Amin Moghaddas Gholami, Kerstin E. Geillinger, Hannelore Daniel, Bernhard Kuster, Martin Klingenspor

**Affiliations:** ^1^ Chair of Molecular Nutritional MedicineTechnische Universität MünchenElse Kröner Fresenius Center for Nutritional MedicineFreising‐WeihenstephanGermany; ^2^ Z I E L ‐ Research Center for Nutrition and Food SciencesTechnische Universität MünchenFreising‐WeihenstephanGermany; ^3^ Chair of Proteomics and BioanalyticsTechnische Universität MünchenBavarian Biomolecular Mass Spectrometry CenterFreising‐WeihenstephanGermany; ^4^ Nutritional PhysiologyTechnische Universität MünchenFreising‐WeihenstephanGermany

**Keywords:** Age, diet‐induced obesity, fatty acid oxidation, mitochondria, nonalcoholic fatty liver disease, proteomics

## Abstract

Nonalcoholic fatty liver disease (NAFLD) is a major health burden in the aging society with an urging medical need for a better understanding of the underlying mechanisms. Mitochondrial fatty acid oxidation and mitochondrial‐derived reactive oxygen species (ROS) are considered critical in the development of hepatic steatosis, the hallmark of NAFLD. Our study addressed in C57BL/6J mice the effect of high fat diet feeding and age on liver mitochondria at an early stage of NAFLD development. We therefore analyzed functional characteristics of hepatic mitochondria and associated alterations in the mitochondrial proteome in response to high fat feeding in adolescent, young adult, and middle‐aged mice. Susceptibility to diet‐induced obesity increased with age. Young adult and middle‐aged mice developed fatty liver, but not adolescent mice. Fat accumulation was negatively correlated with an age‐related reduction in mitochondrial mass and aggravated by a reduced capacity of fatty acid oxidation in high fat‐fed mice. Irrespective of age, high fat diet increased ROS production in hepatic mitochondria associated with a balanced nuclear factor erythroid‐derived 2 like 2 (NFE2L2) dependent antioxidative response, most likely triggered by reduced tethering of NFE2L2 to mitochondrial phosphoglycerate mutase 5. Age indirectly influenced mitochondrial function by reducing mitochondrial mass, thus exacerbating diet‐induced fat accumulation. Therefore, consideration of age in metabolic studies must be emphasized.

## Introduction

The liver is a central organ in systemic lipid homeostasis. Hepatocytes take up and oxidize lipids, but also synthesize fatty acids de novo. An imbalance of these pathways results in the accumulation of triglycerides, which is the hallmark of nonalcoholic fatty liver disease (NAFLD) (Kawano and Cohen 2013). Mitochondria and endoplasmic reticulum are the major organelles for fatty acid metabolism in mammalian cells. Mitochondria break down fatty acids by beta‐oxidation to fuel oxidative phosphorylation (Kawano and Cohen 2013) and closely cooperate with the endoplasmic reticulum in the synthesis of complex lipids (Rieusset 2011). Mitochondria are not only the prime site of fatty acid oxidation, but also the major source of superoxide. Oxidative phosphorylation operates at the price of superoxide production due to electron leakage in the electron transport system generating multiple forms of reactive oxygen species (ROS) (Brand, in press). In response to increased superoxide production, the nuclear factor erythroid‐derived 2 like 2 (NFE2L2, also known as NRF2) becomes one key regulator of compensatory gene expression to induce cellular ROS defense systems. Under basal conditions NFE2L2 is retained in the cytosol, partly associated with mitochondria, but translocates to the nucleus upon interaction with ROS or oxidized lipid species (Vomhof‐Dekrey and Picklo 2012).

Mitochondrial function and biogenesis decline with age and have been connected to the pathology of various age‐related diseases (Sun et al. 2016). Nutritional challenges, like high dietary fat intake, lead to increased mitochondrial ROS production eventually overwhelming cellular detoxification systems and causing oxidative damage. The “two‐hit hypothesis” of hepatic steatosis and the concurrent increase in oxidative damage links mitochondria closely to the development of NAFLD and the progression toward nonalcoholic steatohepatitis (Paradies et al. 2014).

In modern societies NAFLD is strongly associated with obesity, insulin resistance and type 2 diabetes (Perry et al. 2014). The development of NAFLD has been frequently studied in animal models exposed to high fat diets (Begriche et al. 2013; Kakimoto and Kowaltowski 2016). Several global omics‐based studies have addressed the molecular aspects of fatty liver development in diet‐induced obese rodent models and diabetic mice (Baiges et al., 2010; Xie et al., 2010; Zhang et al. 2010; Bondia‐Pons et al., 2011; Kirpich et al., 2011; Oh et al., 2011; Rubio‐Aliaga et al., 2011; Almon et al., 2012; Midha et al., 2012; Kim et al., 2013; Benard et al., 2016; Cheng et al., 2016). This led to an emerging interest in the role of mitochondria in NAFLD and nonalcoholic steatohepatitis (Guo et al., 2013; Thomas et al., 2013; Li et al., 2014; Nesteruk et al., 2014). Most of these studies investigated the development of fatty liver in mice at juvenile life stages and clearly demonstrate that fat accumulation in adolescent mice can be induced by high fat diet depending on the fat source and the duration of feeding (Satapati et al., 2012; Fontana et al., 2013; Ludwig et al., 2013; Nakamura and Terauchi, 2013). In humans, the prevalence of metabolic diseases, including NAFLD, increases with age (Sheedfar et al., 2013). Beyond the duration of exposure to obesogenic nutrition, physiological changes known to occur with age promote the susceptibility for hepatic fat accumulation, including reduced mitochondrial fatty acid oxidation. On the cellular level the balance of ROS production, detoxification and repair mechanisms deteriorates with age resulting in oxidative damage (Sheedfar et al., 2013).

Mice of the inbred strain C57BL/6, when fed a regular low fat chow diet, exhibit normal hepatic triglyceride content until the age of 12 months (Sheedfar et al., 2014), but develop hepatic steatosis at 18 months (Xiong et al., 2014). Thus, susceptibility for NAFLD increases with age. In response to high fat diet feeding for a fixed period of 16 weeks, however, a recent study found the same level of hepatic steatosis in young and in old mice, with diet‐induced steatohepatitis in the older mice (Fontana et al., 2013). The lack of an age effect on hepatic steatosis was possibly due to the long duration of the dietary intervention. In our effort to trace the early molecular events that facilitate the development of an imbalance in hepatic fatty acid metabolism, we aimed to delineate age‐ and diet‐related effects at an early stage of disease development. A previous study demonstrated that 12 weeks of feeding our control and high fat diet are sufficient to increase hepatic triglyceride content in adolescent mice (Ludwig et al., 2013). To study the effect of age on this process at an even earlier stage, we applied a fixed period of 9 weeks high fat feeding in adolescent (A), young adult (YA), and middle‐aged (MA) mice. We assessed liver triglyceride content, mitochondrial mass, bioenergetics, and superoxide production followed by proteome analysis of mitochondrial preparations. Using these data, we identified links between high fat diet‐ and age‐related changes in mitochondrial function and alterations of the proteome profile.

## Materials and Methods

### Mice

For all experiments male C57BL/6J mice were kept under standard conditions (12 h/12 h light/dark cycle and room temperature) with food and water at libitum in a specific pathogen‐free facility. All protocols for animal experiments comply with the EU directives as realized in the German guidelines for animal care and were approved by the Department of Veterinary Affairs of the Government of Upper Bavaria (Regierung von Oberbayern), Germany (AZ 55.2‐1‐54‐2531‐87‐13).

#### Feeding experiment

Mice of 8 weeks of age (adolescent, A), 16 weeks of age (young adult, YA), and 52 weeks of age (middle‐aged, MA) were included in the feeding trials. Customized control (S5745‐E712) and high fat diet (S5745‐E702, ssniff Spezialdiäten GmbH, Soest, Germany) of defined composition (Table 1) were fed for 9 weeks as described previously (Kless et al., 2015). Body composition was assessed by low‐resolution nuclear magnetic resonance (MiniSpec md series, Bruker Corporation, Billerica, MA). Mice were fasted for 6 h, killed by CO_2_ exposure and exsanguinated prior to dissection of liver. Plasma was prepared and frozen.

**Table 1:** Composition of experimental diets

Ingredient	Control diet	High fat diet
Protein (kJ%)	23.0	18.0
Carbohydrates (kJ%)	65.0	34.0
Fat (kJ%)	12.0	48.0
Energy content (kJ/g)	15.5	22.7
Casein (wt%)	24.0	24.0
Corn starch (wt%)	45.9	26.7
Sucrose (wt%)	5.0	5.0
Maltodextrin (wt%)	5.6	5.6
Soy oil (wt%)	5.0	5.0
Palm oil (wt%)	–	20.0
Cellulose (wt%)	5.0	5.0
Mineral mixture (wt%)	6.0	6.0
Vitamin mixture (wt%)	1.2	1.2

#### Glucose tolerance and fasting insulin levels

Mice were fasted for 6 h before the intraperitoneal glucose tolerance test and received 2.5 mg glucose per g lean mass, as recommended for obese mouse models, when experimental groups differ considerably in body fat mass (Rozman et al., 2015). Blood glucose was monitored using a commercial hand‐held glucometer (FreeStyleLite, Abbott Diabetes Care Inc., Alameda, CA). Fasting glucose was determined before glucose injection. As an index for glucose tolerance the total area‐under‐the‐curve was calculated (Rozman et al., 2015). Fasting insulin was analyzed in plasma using a kit (Ultrasensitive Mouse Insulin Elisa, Mercodia AB, Uppsala, Sweden) according to the manufacturer's instructions.

### Characterization of the liver

#### Triglyceride content

Triglycerides were extracted from frozen ground liver tissue as described elsewhere (Keipert et al., 2014) and quantified using a commercial assay (Serum Triglyceride Determination Kit; Sigma Aldrich, St. Louis, MO).

#### Citrate synthase activity and hepatic mitochondrial mass

Frozen ground liver tissue was homogenized in buffer (50 mmol/L Tris, 1 mmol/L EDTA, pH 7.4 at RT, all Carl Roth GmbH, Karlsruhe, Germany) using a speed mill. Supernatant was collected after centrifugation (13,000*g*, 10 min, 4°C) and protein concentration was determined according to Bradford. For CS activity measurement the homogenate was diluted in assay buffer (100 mmol/L Tris, 1 mmol/L EDTA, 1 mmol/L MgCl_2_, pH 8.2, all Carl Roth, and freshly added (final concentration) DTNB (100 μmol/L), Acetyl‐CoA (300 μmol/L) and oxaloacetate (50 mmol/L), all Sigma Aldrich). CS activity is proportional to the reaction of DTNB with free thiol‐groups, detected at 412 nm. Enzyme activity in isolated mitochondria was detected accordingly. Mitochondrial mass was determined as described previously (Raffaella et al., 2008).

### Mitochondrial Bioenergetics and ROS production

#### Isolation of liver mitochondria

Liver mitochondria were isolated from freshly excised liver as described elsewhere (Schulz et al., 2013) with minor modifications. Briefly, the minced and rinsed tissue was homogenized with five strokes in a glass homogenizer with teflon pestle (Sartorius, Göttingen, Germany), twice centrifuged (800*g*, 10 min, 4°C) reusing the supernatant. Upon pelleting a crude mitochondrial fraction (9000*g*, 10 min, 4°C), the pellet was dispersed on a Percoll (GE Healthcare, Fairfield, CT) density‐gradient and centrifuged (4500*g*, 30 min, 4°C). Pellets of mitochondrial protein were washed twice (9000*g*, 10 min, 4°C) and protein was quantified according to Bradford.

#### Oxygen consumption

Oxygen consumption of mitochondria (15 μg/mL) diluted in respiration buffer (120 mmol/L KCl, 5 mmol/L KH_2_PO_4_, 3 mmol/L HEPES, 1 mmol/L EGTA, all Carl Roth GmbH, with freshly added 0.3% fatty acid free BSA from Sigma Aldrich; pH 7.2) with succinate/rotenone (10 mmol/L/8 μmol/L) and subsequent injection of ADP (6 mmol/L) was recorded using the Seahorse XF 96 Analyzer (Agilent Technologies, Santa Clara, CA). Palmitoylcarnitine/malate (40 μmol/L/5 mmol/L) was tested as substrate using equivalent conditions in a Clarktype electrode (Rank Brothers Ltd, Cambridge, UK). Substrates and ADP were purchased from Sigma Aldrich. In the presence of unlimited substrate the respiratory control ratio (RCR) was calculated as the ratio of oxygen consumption rates in the presence and absence of ADP. In mitochondrial preparations RCR is an index for the capacity of mitochondria to produce ATP by oxidative phosphorylation and the coupling efficiency (Perry et al., 2013).

#### Proton leak measurements

Mitochondrial leak respiration and membrane potential were recorded simultaneously as described before (Jastroch et al., 2012). Briefly, rotenone (8 μmol/L), oligomycin (1 μg/mL), and nigericin (0.15 μmol/L), all Sigma Aldrich, were added to isolated mitochondria (0.4 mg/mL) in respiration buffer, before leak respiration was initiated with succinate (10 mmol/L). Malonate titration (0.2–4.2 mmol/L) competitively blocked leak respiration.

#### ROS measurement

Superoxide anion generation, referred to in the following as ROS production, was assessed indirectly by monitoring the hydrogen peroxide‐dependent formation of fluorescent resorufin from Amplex Red (50 μmol/L, Invitrogen, Carlsbad, CA) mediated by horseradish peroxidase (6 U/mL, Sigma Aldrich) as described previously (Grunz et al., 2012). Mitochondria were diluted to 0.1 mg/mL in respiration buffer without BSA. Fluorescence was recorded over 10 min before and 30 min after addition of succinate (10 mmol/L) and rotenone (2 μmol/L).

### Proteome analysis of isolated mitochondria

#### LC‐MS/MS measurement

Pools of isolated mitochondria from five mice of each age and diet group were created. A total of 100 μg protein from each pool was used for three technical replicates. Proteins were digested by the FASP approach (Wisniewski et al., 2009) and the resulting peptides were fractionated by strong anion exchange stage tips prior to nano LC‐MS/MS analysis on an Eksigent nanoLC‐Ultra 1D+ (Eksigent, Dublin, CA) coupled to an Orbitrap Elite mass spectrometer (Thermo Scientific, Bremen, Germany) (Pachl et al., 2013). Peptides were delivered to a trap column (100 μm × 2 cm, packed in‐house with Reprosil‐Pur C18‐AQ 5 μm resin, Dr. Maisch, Ammerbuch, Germany) at a flow rate of 5 μL/min in 100% solvent A (0.1% formic acid in HPLC grade water). After 10 min of loading and washing, peptides were transferred to an analytical column (75 μm × 40 cm, packed in‐house with Reprosil‐Pur C18‐Gold, 3 μm resin, Dr. Maisch) and separated using a 110 min gradient from 7% to 35% of solvent B (0.1% formic acid in acetonitrile) at 300 nL/min flow rate. The Orbitrap Elite was operated in data‐dependent mode, automatically switching between MS and MS2. Full scan MS spectra were acquired in the Orbitrap at 30,000 (m/z 400) resolution after accumulation to a target value of 1,000,000. Tandem mass spectra were generated for up to 15 peptide precursors in the orbitrap for fragmentation using higher energy collision‐induced dissociation (HCD) at a normalized collision energy of 30% and a resolution of 15,000 with a target value of 20,000 charges after accumulation for max 100 ms.

#### Peptide and protein identification and quantification

Progenesis software (version 3.1; Nonlinear Dynamics, Newcastle, UK) was used for intensity‐based label‐free quantification. MS/MS spectra were transformed into peak lists and exported to generate Mascot generic files. The Mascot generic files were searched against UniProt mouse (version 26.10.2010; 73,688 sequences) using Mascot (version 2.3.0; Matrix Science, London, UK). Search parameters were as follows: precursor tolerance 10 ppm, fragment tolerance 0.02 Da, full tryptic specificity with up to two missed cleavage sites, miss‐assignment of the monoisotopic peak to the first 13C peak, fixed modification of carbamidomethylation of cysteine residues and variable modification of N‐terminal protein acetylation and methionine oxidation. Search results for spectrum to peptide matches were exported in.xml format and then imported into Progenesis software to enable the combination of peptide quantification and identification. Peptides with mascot ion scores ≤32 (*P* = 0.05 identity threshold) were filtered out, and only unique peptides for corresponding proteins were used for identification and quantification. For qualitative analysis, the database search results were imported into Scaffold (version 3.6.2; Proteome Software, Portland, OR) for further evaluation.

#### Pathway analysis

Statistical analysis was achieved using “R” (version 2.12.1) (R Development Core Team, 2011). Differential expression of samples was assessed with a moderated linear model using the limma package (Smyth, 2004) in Bioconductor (Gentleman et al., 2004). K‐means clustering was used to cluster “control” versus “high fat” time points and further analyses were conducted in Genomatix Pathway Analysis (GePS, version v2.70226; Munich, Germany) and QIAGEN's Ingenuity Pathway Analysis (IPA, version 1642223; QIAGEN, Redwood City, CA).

### Western blot analysis

Proteins were separated by SDS‐PAGE, transferred to a nitrocellulose membrane and blocked in BSA. Primary antibodies were diluted as follows: NFE2L2 (H300) 1:2500 (sc‐13032; Santa Cruz Biotechnology, Inc., Dallas, TX), PGAM5 1:3000, CS 1:5000 and SOD2 1:10000 (ab126534, ab16956, ab96600; Abcam plc, Cambridge, UK). IR dye‐coupled secondary antibodies were detected in a fluorescence scanner (Odyssey Scanner; LI‐COR Biosciences, Lincoln, NE). Quantitative analysis was performed using ImageJ software (Schneider et al., 2012).

### Statistics

Apart from the proteome data, statistics were assessed using two‐way ANOVA (SigmaPlot 12.5; Systat Software Inc., San Jose, CA) for significant effects of age and diet. In case of deviations from normal distribution, data were log transformed [log(*x *+ 1)] prior to analysis. Outliers were defined by Grubb's test and removed. Significance of diet‐ or age‐related effects was further evaluated by post hoc pairwise comparisons using the Holm–Sidak to adjust for multiple testing with high statistical power. Graphs and tables contain mean values ±standard deviation (SD). ANCOVA was performed to adjust for the effects of body mass as a covariate (Packard and Boardman, 1999). Paired, two‐tailed Student's t‐test was applied for testing the pools of mitochondrial protein upon immunoblot detection and quantification.

## Results

### Diet and age influenced body mass development and glucose tolerance

At the onset of the high fat diet feeding trial, adolescent (A) mice weighed less than young adult (YA) and middle‐aged (MA) mice (Table 2; *P* < 0.001 for A vs. YA and A vs. MA, respectively). At the end of the trial after 9 weeks, final body mass and fat mass were significantly increased by age and diet, with a significant age × diet interaction (Table 2). The latter effect was caused by the augmentation of the high fat diet‐induced increase in body mass and body fat mass with age. Changes in body mass were mainly due to changes in fat mass, as high fat diet did not influence lean mass. We therefore addressed whether the differences in body fat mass scaled in proportion using final body mass as a covariate in ANCOVA. The diet effect persisted after adjustment of body fat mass to body mass, whereas the age effect was abolished (Table 2). The age effect in final lean mass also disappeared after ANCOVA analysis. Taken together, absolute changes in body mass and body composition were significantly affected by age, but on the relative level diet‐induced obesity was proportional to body mass, and not aggravated with age.

**Table 2:** Body composition and glucose metabolism of three age groups of mice after 9 weeks of high fat diet feeding. At the onset of the dietary intervention mice were either at the age of 8 weeks (adolescent), 16 weeks (young adult), or 52 weeks (middle‐aged)

	Adolescent		Young adult		Middle‐aged		Two‐way ANOVA		
	Control	High fat	Control	High fat	Control	High fat	*P*(Age)	*P*(Diet)	*P*(AgeDiet)
Body mass _initial_	21.4 ± 0.9	21.2 ± 0.9	26.6 ± 0.8^1^	26.1 ± 0.9^1^	30.2 ± 0.7^1,2^	30.2 ± 1.1^1,2^	<0.001	n.s.	n.s.
Body mass _final_	24.7 ± 0.7	29.5 ± 1.0^3^	27.8 ± 0.7^1^	36.0 ± 2.4^1,3^	30.7 ± 0.9^1,2^	43.7 ± 2.0^1,2,3^	<0.001	<0.001	<0.001
Body mass gain	3.2 ± 0.7	8.3 ± 1.1^3^	1.2 ± 0.7	9.9 ± 2.3^3^	0.4 ± 0.6^1^	13.5 ± 2.4^1,3^	n.s.	<0.001	<0.001
Fat mass _initial_	1.7 ± 0.2	1.8 ± 0.2	2.9 ± 0.5^1^	2.7 ± 0.1^1^	3.1 ± 0 5^1^	3.4 ± 0.4^1,2^	<0.001	n.s.	n.s.
Fat mass _final_	2.1 ± 0.3	6.6 ± 0.8^3^	3.1 ± 0.4	11.8 ± 1.7^1,3^	3.5 ± 0.6	17.3 ± 1.2^1,2,3^	<0.001	<0.001	<0.001
Fat mass gain	0.4 ± 0.2	4.9 ± 0.8^3^	0.2 ± 0.3	9.1 ± 1.8^1,3^	0.4 ± 0.8	13.9 ± 1.2^1,2,3^	<0.001	<0.001	<0.001
Lean mass _initial_	16.1 ± 0.9	15.7 ± 0.8	19.6 ± 0.4^1^	19.4 ± 0.6^1^	22.7 ± 0.8^1,2^	22.3 ± 0.7^1,2^	<0.001	n.s.	n.s.
Lean mass _final_	18.3 ± 0.8	19.5 ± 0.6	20.2 ± 0.6^1^	19.8 ± 0.6	21.7 ± 0.5^1,2^	21.2 ± 0.7^1,2^	<0.001	n.s.	0.003
Lean mass gain	2.2 ± 0.3	3.8 ± 0.6	0.6 ± 0.6^1^	0.4 ± 0.3^1^	−1.0 ± 1.1^1,2^	−1.1 ± 1.4^1,2^	<0.001	n.s.	0.02
Adjusted fat mass^4^	2.7 ± 0.4	11.6 ± 1.0^3^	3.0 ± 0.3	12.1 ± 0.5^3^	2.9 ± 2.9	12.0 ± 1.5^3^	n.s.	<0.001	n.s.
Adjusted lean mass^4^	20 ± 0.5	20.4 ± 0.5	20.2 ± 0.4	19.8 ± 0.5	20.0 ± 0.7	20.3 ± 0.7	n.s.	n.s.	n.s.
Fasting Glc _Final_	7.7 ± 1.7	10.4 ± 0.9^3^	9.4 ± 0.5^1^	10.1 ± 1.4	7.6 ± 0.4^2^	9.3 ± 1.1^3^	0.03	<0.001	n.s.
Fasting Insulin	0.6 ± 0.3	1.3 ± 0.6^3^	0.6 ± 0.3	1.5 ± 0.5^3^	1.0 ± 0.4	4.2 ± 1.1^1,2,3^	<0.001	<0.001	<0.001
Total AUC^5^	177 ± 39	275 ± 32^3^	229 ± 33	321 ± 63^3^	227 ± 35	317 ± 23^3^	0.007	<0.001	n.s.
Liver mass	1.3 ± 0.1	1.2 ± 0.2	1.2 ± 0.1	1.5 ± 0.3	1.6 ± 0.1^1,2^	1.7 ± 0.2^1^	<0.001	n.s.	n.s.
Adjusted liver mass^4^	1.5 ± 0.1	1.2 ± 0.2	1.3 ± 0.1	1.4 ± 0.3	1.6 ± 0.1	1.4 ± 0.2	n.s.	n.s.	n.s.

*n* = 6 per diet and age group. Data are represented as means ± SD. Two‐Way ANOVA statistics for *P*(Age) – age effect, *P*(Diet) – diet effect, and *P*(AgeDiet) – interaction. n.s., not significant (*P* ≥ 0.05).

Results of post hoc testing are indicated by ^1^Significantly different to Adolescent within feeding group; ^2^Significantly different to Young Adult within feeding group; ^3^Significantly different to age‐matched control group.

^4^Adjustment for final body mass as covariate by ANCOVA.

^5^AUC – area‐under‐the‐curve of ipGTT in (min × g × L^−1^). Body mass, fat mass and lean mass, initial and final, body mass gain, fat mass gain, lean mass gain, adjusted fat mass, adjusted lean mass, liver mass, and adjusted liver mass – (g); fasting glucose – (mmol/L); fasting insulin – (ng × mL^−1^).

Mean fasting glucose levels were elevated in all high fat diet‐fed groups compared to their age‐matched controls (Table 2) with no systematic effect of age. Fasting insulin levels were also increased by high fat diet, with pronounced hyperinsulinemia in MA mice (Table 2). Possible alterations in glycemic control were further assessed by glucose tolerance tests. All high fat‐fed mice displayed decreased glucose tolerance, as depicted from the increased area‐under‐the‐curve. Glucose tolerance was impaired by age in YA and MA mice as compared to A mice, when age groups were analyzed irrespective of diet (*P*(A, YA) < 0.05, *P*(A, MA) < 0.05, *P*(YA, MA) n.s.).

### High fat diet stimulated hepatic fat accumulation only in YA and MA mice and aggravated age‐related reduction in mitochondrial mass

**Figure 1:**
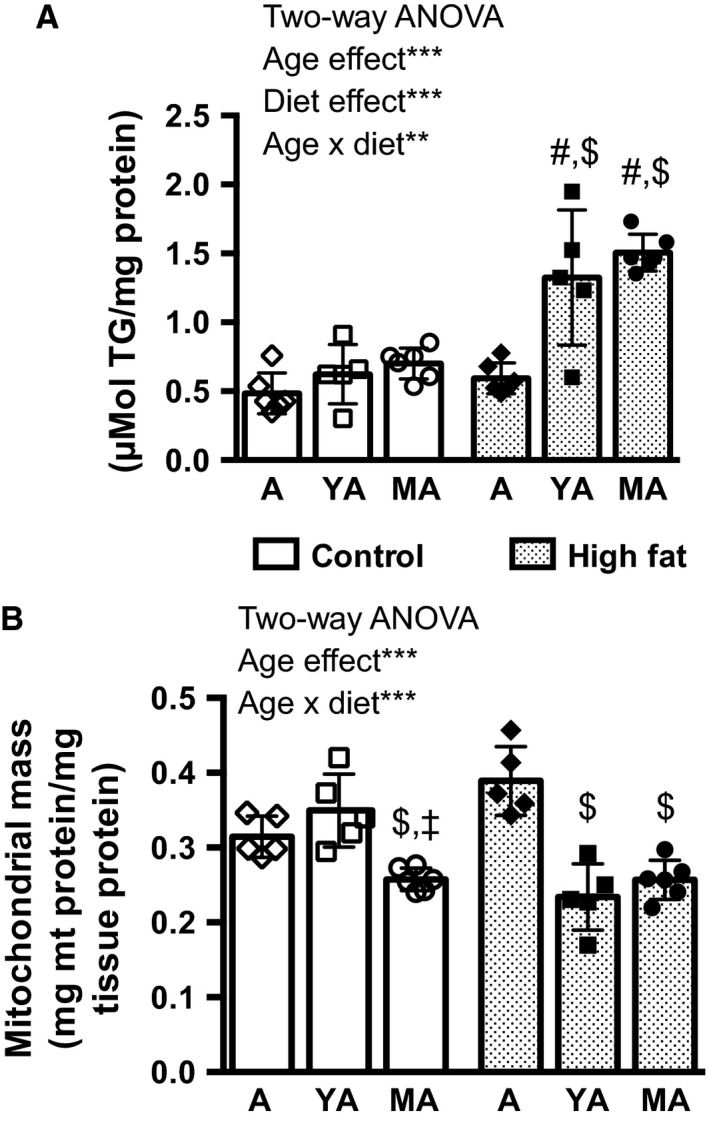
(A) Hepatic triglyceride (TG) content and (B) mitochondrial (mt) mass in adolescent (A), young adult (YA), or middle‐aged (MA) mice after 9 weeks of either control diet or high fat diet feeding. *n* = 5–6 per age and diet group, Two‐Way ANOVA effects are indicated by asterisks: **P < 0.01; ***P < 0.001. Results of post hoc testing are indicated by ^#^Significantly different to age‐matched control group; ^$^Significantly different to A within feeding group; ^‡^Significantly different to YA within feeding group. Data are represented as means ± SD.

Liver mass increased with age but was not affected by diet. The effect of age was due to increased body mass in older mice, and eliminated after adjusting for the final body mass by ANCOVA (Table 2). Hepatic triglyceride content, however, increased with age. A significant age × diet interaction was detected (Fig. 1A). In mice fed a high fat diet, we observed increased lipid accumulation in YA and MA compared to A mice, but also compared to age‐matched controls (Fig. 1A).

Liver mitochondrial mass was determined based on citrate synthase (CS) activities in crude liver homogenates and in isolated mitochondria (Raffaella et al., 2008). On control diet, MA mice had less mitochondrial mass than A and YA mice (Fig. 1B). On high fat diet, however, not only MA, but also YA mice had reduced mitochondrial mass compared to A mice (Fig. 1B). Statistics confirmed age × diet interaction for mitochondrial mass. Taken together both, age and high fat diet, impacted on liver composition.

### High‐fat diet reduced respiration rates on fatty acids in hepatic mitochondria and increased ROS production rates

To identify the impact of high fat diet feeding on mitochondrial function, we subjected isolated liver mitochondria to bioenergetic profiling. Mitochondrial oxidative phosphorylation rates, proton leak, and ROS measurements were conducted. Using succinate as a substrate, mitochondrial respiration rates were comparable in high fat‐fed and age‐matched control‐fed mice (Table 3). However, when oxidizing palmitoylcarnitine, liver mitochondria of high fat‐fed mice in the phosphorylating state (+ADP) consumed less oxygen than control‐fed mice (Table 3). The respiratory control ratio (RCR) describes the ability of mitochondria to increase oxygen consumption for ATP production when ADP is not limiting. Although total respiration rates increased with age, the RCR declined. No effect of diet was observed with succinate as substrate. Upon initiation of fatty acid‐driven oxidative phosphorylation, RCR was markedly reduced in mitochondria of MA mice fed high fat diet compared to high fat‐fed YA mice (Table 3).

**Table 3:** Oxygen consumption in isolated liver mitochondria of adolescent, young adult, and middle‐aged mice

Oxygen consumption^4^		Adolescent		Young adult		Middle‐aged		Two‐way ANOVA		
		Control	High fat	Control	High fat	Control	High fat	*P*(Age)	*P*(Diet)	*P*(AgeDiet)
Succ	−ADP	13 ± 6	15 ± 7	27 ± 8	21 ± 5	50 ± 16^1^ ^,^ ^2^	58 ± 19^1^ ^,^ ^2^	<0.001	n.s.	n.s.
	+ADP	47 ± 19	42 ± 15	76 ± 28	57 ± 10	127 ± 23^1^ ^,^ ^2^	155 ± 46^1^ ^,^ ^2^	<0.001	n.s.	n.s.
RCR		3.8 ± 0.3	3.0 ± 0.7	2.7 ± 0.5^1^	2.7 ± 0.3	2.7 ± 0.6^1^	2.7 ± 0.2	0.002	n.s.	n.s.
PC/Mal	−ADP	12 ± 3	10 ± 2	16 ± 4	14 ± 4^1^	22 ± 5^1^ ^,^ ^2^	20 ± 4^1^ ^,^ ^2^	<0.001	n.s.	n.s.
	+ADP	69 ± 15	47 ± 7^3^	83 ± 11	82 ± 25^1^	95 ± 23^1^	75 ± 9^1^ ^,^ ^3^	0.002	0.02	n.s.
RCR		6.0 ± 1.0	4.9 ± 1.0	5.4 ± 1.5	5.8 ± 1.4	4.3 ± 0.5^1^	3.8 ± 0.7^2^	0.002	n.s.	n.s.

RCR, respiratory control ratio.

Results of post hoc testing are indicated by ^1^Significantly different to Adolescent within feeding group;

^2^ignificantly different to Young Adult within feeding group;

^3^Significantly different to age‐matched control group.

^4^nmol O_2_ × mg^−1^ × min^−1^, Succ, succinate/rotenone, PC/Mal, palmitoylcarnitine/Malate. Data are represented as means ± SD; *n* = 5–6 per group; Two‐Way ANOVA statistics for *P*(Age) ‐ age effect, *P*(Diet) ‐ diet effect, and *P*(AgeDiet) ‐ interaction.

**Figure 2:**
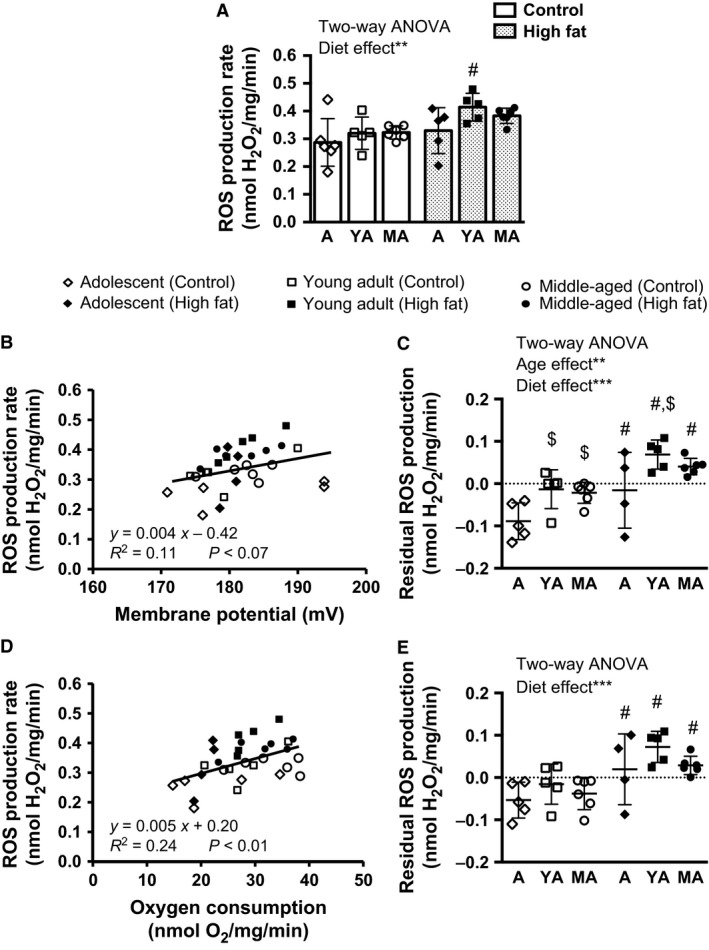
(A) Mitochondrial ROS production rates, monitored as H_2_O_2_ released by liver mitochondria, isolated from adolescent (A), young adult (YA), and middle‐aged (MA) mice fed either a high fat or control diet for 9 weeks. Mitochondrial respiration was initiated with succinate/rotenone in absence of ADP. (B) Scatter plot analysis of mitochondrial ROS production rates plotted versus membrane potential or (D) oxygen consumption rate during leak respiration. Based on the regression equations shown in (B) and D), the respective residual ROS production rates were calculated in (C) and (E). *n* = 4–6 per age and diet group. Two‐Way ANOVA statistics are indicated with **P < 0.01; ***P < 0.001; results of post hoc testing are indicated by ^#^Significantly different to age‐matched control group; ^$^Significantly different to A within feeding group. Data are represented as means ± SD.

Proton leak kinetics, as assessed by measurements of leak respiration and membrane potential in the absence of ATP synthesis, confirmed an age‐dependent decrease in the efficiency to build up the proton gradient (*P* < 0.05 for age effect; data not shown), as reported previously as an age‐related trait (Lopez‐Otin et al., 2013). High fat diet feeding did not affect proton leak kinetics (data not shown). Under identical assay conditions, however, the rate of mitochondrial superoxide anion generation, hereafter annotated in text and figures as ROS production, was increased by high fat diet feeding (Fig. 2A). Post hoc testing confirmed increased ROS production by high fat diet feeding in the YA group and a trend in the MA group (*P* = 0.09).

As membrane potential and oxygen consumption impact on ROS production rates, we adjusted for these parameters using regression analysis (Fig. 2B and D). Data points depicting negative residuals for ROS production reside below the regression line, which is seen in most control mice (open symbols). Almost all high fat mice had positive residuals lying above this line (closed symbols).

Therefore, liver mitochondria of high fat‐fed mice produce more superoxide anions than control‐fed mice, irrespective of the given membrane potential or oxygen consumption (Fig. 2C and E). The general diet effect was confirmed in all age groups by post hoc testing between control and high fat‐fed mice (*P* < 0.05 in A and MA mice, respectively, *P* < 0.01 for YA mice). After adjustment for membrane potential, age affected residual ROS production rates, which were elevated in YA and MA control mice compared to A control mice, whereas within the high fat‐fed mice ROS was higher in YA compared to A and MA mice (Fig. 2C). As the residual ROS production rate, adjusted for variation in membrane potential, was elevated in control YA and MA as well as in high fat‐fed YA mice compared to A mice, this hints toward a substantial higher ROS production rate from liver mitochondria of YA and MA mice which may contribute to the bioenergetic imbalance driving hepatic fat accumulation.

### Highly pure isolates of liver mitochondria were subjected to proteome analysis

**Figure 3:**
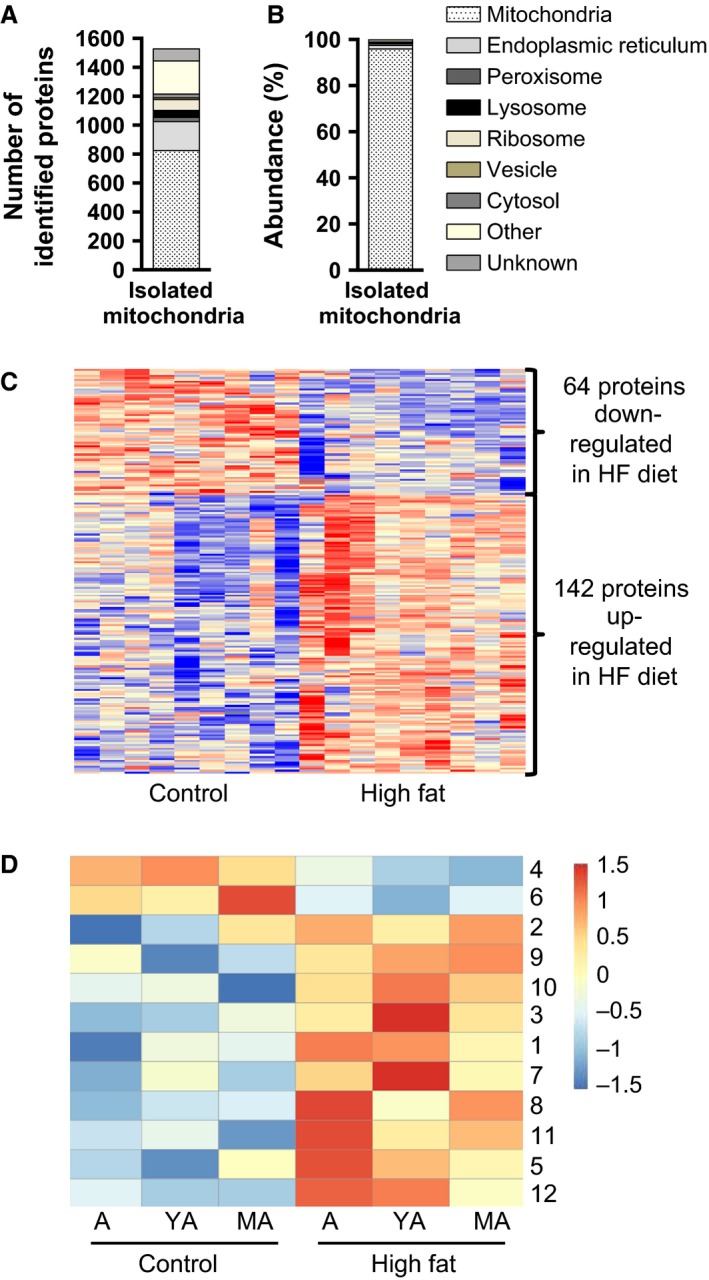
(A) Number of all different identified proteins and attributed subcellular localization, (B) Total abundance of proteins identified and attributed to subcellular localization, (C) Hierarchical clustering of proteins significantly regulated by high fat (HF) diet, and (D) age‐related clustering of proteins regulated by high fat diet. A, adolescent; YA, young adult; MA, middle‐aged

In the next step, we sought to identify proteins linked to diet‐ and age‐associated changes in mitochondrial function. For each age and diet group pools of isolated mitochondria were profiled for changes in protein levels. Proteome analysis confirmed the quality of the mitochondrial isolation. In total, 1527 distinct proteins were identified, of which 824 localized to mitochondria, as annotated in the databases Genomatix (GePS) and MitoCarta (Pagliarini et al., 2008) (Fig. 3A). Taking into account protein abundance, these 824 proteins made up 95% of the total protein in our isolates. The remaining ~700 proteins, representing 5% of the total protein, localized to the endoplasmic reticulum, peroxisomes, lysosomes, and other subcellular compartments or structures (Fig. 3B).

In response to high fat diet, 206 proteins were found differentially expressed as illustrated in a heat map (Fig. 3C). Within this subset of 206 regulated proteins, the subcellular distribution was similar compared to the entity of 1527 proteins identified. The abundance of diet‐responsive proteins was further analyzed with respect to the effect of age and organized in similar age‐related patterns according to k means algorithm (Fig. 3D). A complete list of regulated proteins is provided as supplemental data (Table S1 ‐ Regulated proteins by diet; normalized intensity for all identified proteins and replicates is provided in Table S2).

### Fatty acid oxidation and mitochondrial‐related pathways

Within the subset of 206 differentially expressed proteins, we identified 86 mitochondrial proteins by gene ontology and literature mining and inspected their known functions (Table 4). In several categories of biological functions, we observed up and downregulation of proteins, whereas in some categories only up or downregulation was seen. Among the latter, identified proteins are involved in fatty acid handling, for example, CoA activation (ACSM3, ACSS3, ACSF2, SLC27A2), ketone body metabolism (ACAT1), TCA cycle (SUCLA2, SUCLG2), and sphingolipid metabolism (PSAP). Fatty acid oxidative enzymes acyl‐CoA dehydrogenases (ACADS and ACAD10), which catalyze the first dehydrogenation of fatty acids (Wanders et al., 1999), and propionyl‐CoA carboxylase (PCCA), the substrate of which arises from odd‐numbered chain fatty acids and branch‐chained amino acids (Desviat et al., 2004), were upregulated. Trifunctional protein (HADHA) catalyzes three consecutive steps of the fatty acid oxidation cycle with specificity for long and medium chain fatty acids (Wanders et al., 1999) and was found reduced in livers of high fat‐fed mice by 10% (Table 4, supplemental Table S1). EHHADA and CROT, two enzymes allocated to mitochondria and peroxisomes, were reduced after high fat feeding (Table 4). Another peroxisomal enzyme for fatty acid degradation, ACOX2, is downregulated in high fat‐fed mice (supplemental Table S1), suggesting a reduction in the peroxisomal contribution to fatty acid utilization.

**Table 4:** Overview and gene ontologies in respect to “Biological Function” of 86 mitochondrial proteins identified to be regulated by diet

Mitochondrial proteins		
GO biological function	Protein name	
	DOWN	UP
Heme biosynthesis	FECH, PPOX	
Programmed cell death	PGAM5	
Nucleotide biosynthesis	DTYMK	
Peptidases	CTSA, CTSB	
Purine metabolism	UOX, 1190003J15Rik	
Sphingolipid metabolic process	PSAP	
Aminotransferase	AGXT	OAT, AGXT‐2, GOT2
Amino acid catabolic pathway	AASS, GCAT	BCKDH, BCKDHB, BCKDHA, MCCC1, DMGDH, GCDH
Fatty acid beta‐oxidation	HADHA, CROT, EHHADH	ACAD10, ACADS, ACAA2, CYB5A, PCCA
Hydroxyacid‐oxoacid transhydrogenase	ADHFE1	ADHFE1
Inner mitochondrial membrane organization	MINOS1	OPA1, LETM1
Mitochondrial translation	MRPL24, MRPS5	MRPS7
Protein import	SAMM50	PMPCA
Respiratory chain	CYTCS, NDUFS6, NDUFAF4, ATP5D	NDUFAF1
Response to oxidative stress	CAT, SOD1	MSRA, DNAJC11, MGST1
Sulfur metabolism	ETHE1	SUOX, SQRDL
Urea cycle	CPS1	OTC
Acyl‐CoA ligase activity		ACSM3, ACSS3, ACSF2, SLC27A2
Calcium homeostasis		RMDN3 (Ptpip51)
Cellular aldehyde metabolic process		ALDH3A2
Glutamate/glutamine metabolism		GLUL, GLUD1
Ketone body production		ACAT1
Oxidoreductase ‐ heme binding		CYP22D2, AKR7A5, FDXR
TCA cycle		SUCLA2, SUCLG2
Transmembrane transport		ABCB7, MTCH1, SLC25A45, SLC25A10, SLC25A13, SLC25A1
Unknown function		MTFR1L, ADCK1, LRRC59, UPF0640, RMDN2, C14orf159, SND1, ISOC2, MMAA, C21orf33, BPHL, HINT2

Mitochondrial proteins involved in the electron transport chain or ATP synthesis did not show a conclusive regulation pattern (Table 4). Interestingly, SLC25A3 was down‐regulated with age in MA mice compared to YA or A mice (supplemental Table S1‐ Regulated proteins by age). This transporter imports free phosphate for ATP synthesis into the mitochondrial matrix using the proton motive force (Seifert et al., 2015). Otherwise, no mitochondrial pathways stood out as confirmed by the use of GePS and IPA.

### Nonmitochondrial pathways indicate increased protein synthesis, VLDL assembly, and age‐related reduction in lysosomal degradation

Further pathway analysis including all 206 diet‐responsive proteins revealed upregulation of proteins involved in translation or protein processing/glycosylation, EIF2 signaling, and protein processing in the endoplasmic reticulum. Furthermore, phospholipid synthesis and proteins for assembly and transport of lipoproteins were upregulated. Phospholipid synthesis requires cooperation of endoplasmic reticulum and mitochondria (Rieusset, 2011). Levels of proteins known to tether endoplasmic reticulum and mitochondrial membranes were upregulated by high fat diet (supplemental Table S1 – Biological Functions).

Intriguingly, high fat diet feeding caused triglyceride accumulation in the livers of YA and MA mice, but not in A mice (Fig. 1A). We therefore searched the regulatory clusters of the proteome data (Fig. 3D) for correlations with liver triglyceride content. Cluster 4, which comprises proteins downregulated by high fat diet, exhibited the best correlation with hepatic triglyceride content. The abundance of proteins in cluster 4, comprising 14 regulated lysosomal proteins, decreased with age. Regression analysis demonstrated significance for five lysosomal proteins: lysosomal associated membrane protein 2 (LAMP2; *P* = 0.002, *r* = −0.96, *R* ^2^ = 0.93), phospholipase B domain containing 1 (PLBD1; *P* = 0.009, *r* = −0.92, *R* ^2^ = 0.85), cathepsin (CTS) H (*P* = 0.01, *r* = −0.92, *R* ^2^ = 0.84), CTSS (*P* = 0.02, *r* = −0.88, *R* ^2^ = 0.77), CTSA (*P* = 0.02, *r* = −0.87, *R* ^2^ = 0.76) with strong correlation coefficients (*r*) and coefficient of determination (*R* ^2^). Conclusively, downregulation of these lysosomal proteins with age is strongly associated with detrimental accumulation of triglycerides in liver of high fat‐fed mice.

### Increased mitochondrial ROS production is related to a balanced NFE2L2‐dependent response

Liver mitochondria isolated from high fat‐fed mice exhibited increased ROS production. It was therefore of interest that our pathway analysis identified an overrepresentation of enzymes regenerating oxidized molecules in livers of high fat‐fed mice (Table 5). These proteins are targets of NFE2L2‐dependent response to oxidative stress. Beyond these, we identified more differentially regulated proteins in our proteome study reported to be under transcriptional control of NFE2L2 by consulting a published screen for NFE2L2‐regulated genes (Kwak et al., 2003).

**Table 5:** Proteins regulated by NFE2L2 or identified as interaction partners of NFE2L2

Uniprot ID	Protein name	FC	Pathway/Function (Annotation/Reference)	Match of predicted versus observed regulation (Y/N)
B1ATI0	ALDH3A2	2.1	Target of NFE2L2 regulon (Kwak et al., 2003), repair of lipidperoxides (Demozay et al., 2008)	Y
A2AMV3	AKR7A5	1.4	NFE2L2‐mediated oxidative stress response (IPA), repair of lipidperoxides (Li et al., 2006)	Y
Q5EBQ7	MSRA	1.4	Co‐cited transcription factor NFE2L2 (GePS), repair of oxidized cysteine residues (Styskal et al., 2013)	Y
A2A8C9	HSP40 (DNAJC11)	1.4	NFE2L2‐mediated oxidative stress response (IPA)	Y
Q3TXQ6	CAT	0.7	NFE2L2‐mediated oxidative stress response (IPA, GePS)	N
P08228	SOD1	0.6	NFE2L2‐mediated oxidative stress response (IPA, GePS)	N
Q91VS7	MGST1	1.3	NFE2L2‐mediated oxidative stress response (IPA, GePS), repair of lipidperoxides (Kelner et al., 2000)	Y
Q64435	UGT1A6	2.6	Co‐cited transcription factor NFE2L2 (GePS)	Y
O35728	CYP4A14	0.4	Co‐cited transcription factor NFE2L2 (GePS),microsomal FAO (Gambino et al., 2011)	N
Q8VCW9	CYP2A12	1.8	Target of the NFE2L2 regulon^a^	Y
B6VGH4	CYP1A2	1.6	Target of the NFE2L2 regulon^a^	Y
O35490	BHMT	1.9	Target of the NFE2L2 regulon^a^	Y
Q543J0	UOX	0.7	Target of the NFE2L2 regulon^a^	Y
O88833	CYP4A10	0.5	Target of the NFE2L2 regulon^a^, microsomal FAO (Gambino et al., 2011)	N
Q8BJL4	LMAN2	1.6	Target of the NFE2L2 regulon^a^	Y
Q9D8V0‐3	HM13	1.7	Target of the NFE2L2 regulon^a^	Y
O88668	CREG1	0.5	Target of the NFE2L2 regulon^a^	N
Q3TJI8	HSD11B1	2.0	Co‐cited transcription factor NFE2L2 (GePS), inactivates NFE2L2 (Kratschmar et al., 2012)	–
A1A4A7	PGAM5	0.5	Co‐cited transcription factor NFE2L2 (GePS), tethers NFE2L2 to mitochondria (Xue et al., 2015)	–

a Kwak et al. (2003), FC – fold change reflects the mean abundance of high fat‐fed mice divided by the mean abundance of control‐fed mice.

Intriguingly, some classical NFE2L2 targets, like superoxide dismutase 1 (SOD1) and catalase, were downregulated in our proteome dataset, contradicting NFE2L2‐dependent transcriptional control. Further text mining via GePS, however, linked hydroxysteroid (11‐beta) dehydrogenase 1 (HSD11B1) and phosphoglycerate mutase 5 (PGAM5) to NFE2L2, which are both proteins with the potential to modulate NFE2L2 activation. The increased level of HSD11B1 in livers of high fat‐fed mice hints toward inhibition of NFE2L2 (Kratschmar et al., 2012), whereas reduced PGAM5 favors the translocation of NFE2L2 into the nucleus (Xue et al., 2015).

**Figure 4:**
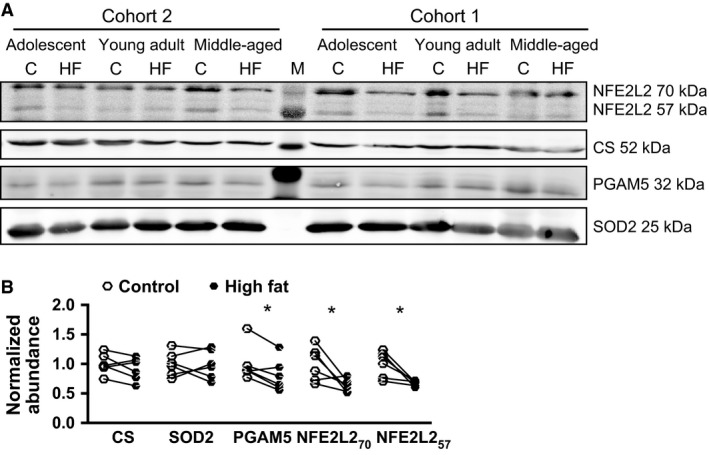
(A) Western blot analysis of isolated mitochondria. Samples of individual mice were pooled per diet and age group. Cohort 2 represents protein pools, which were also subjected to proteome analysis. Cohort 1 is composed of independent experimental groups for the respective ages and diets. Protein abundance was validated for nuclear factor erythroid‐derived 2 like 2 (NFE2L2), Citrate Synthase (CS), phosphoglycerate mutase 5 (PGAM5), and superoxide dismutase 2 (SOD2). (B) Band intensities were quantified and expressed relative to the mean of controls. Data are represented as means ± SD. Statistics were performed using paired, two‐tailed Student's *t*‐test for control (C) and high fat (HF) group of the respective ages per cohort. *P < 0.05, *n* = 6. M, protein ladder.

PGAM5 tethers NFE2L2 via its inhibitor to the outer mitochondrial membrane (Xue et al., 2015). Connecting this to our observation of increased mitochondrial ROS production in livers of high fat‐fed mice, we hypothesized that down‐regulation of PGAM5 in liver mitochondria by a high fat diet is associated with diminished mitochondrial NFE2L2 levels. Protein pools were subjected to Western blot analysis for NFE2L2, PGAM5, CS, and SOD2 (Fig. 4A). Quantification confirmed a significant down‐regulation (*P* < 0.05) of PGAM5 in isolated mitochondria of high fat‐fed mice. In line with our proteome analysis the mitochondrial markers CS and SOD2 were not regulated. Two NFE2L2 bands were detected at 57 kDa and 70 kDa, the latter given by the UniprotKB database. Pertaining to both bands, the predicted down‐regulation of mitochondrial NFE2L2 in high fat‐fed mice was corroborated (Fig. 4B).

Taken together, Western blot analysis confirmed the hypothesis generated by our proteome analysis. The coregulation of PGAM5 and NFE2L2 suggests a mechanistic link between increased mitochondrial ROS production, down‐regulation of PGAM5 and the initiation of an antioxidative response by NFE2L2‐dependent gene expression in livers of high fat‐fed mice.

## Discussion

The association between mitochondrial dysfunction and the development of fatty liver is widely accepted (Begriche et al., 2013). However, the molecular mechanisms responsible for these alterations have not been fully established, despite considerable efforts to understand the causes and consequences of NAFLD including comprehensive omics‐based studies (Xie et al., 2010; Kirpich et al., 2011; Oh et al., 2011; Midha et al., 2012; Thomas et al., 2012, 2013). In this study, we provide bioenergetic analyses of liver mitochondria combined with proteome profiling at a very early stage of high fat diet‐induced fatty liver development in mice. Our study design addressed the possible contributions of age‐related metabolic changes, including increased diet‐induced obesity susceptibility, impaired glucose tolerance and decline of mitochondrial functions.

The fixed period of high fat diet feeding for 9 weeks effectively caused diet‐induced obesity in A, YA, and MA mice. Our analysis of absolute and relative levels of adiposity revealed contrasting outcomes in respect to the effect of age on diet‐induced obesity. Regarding the absolute gains in body mass and body fat the susceptibility for diet‐induced obesity increased with age (MA > YA > A). Body fat mass, however, increased in proportion with body mass. Hyperglycemia and impaired oral glucose tolerance were induced by high fat diet feeding without age‐dependent aggravation, whereas pronounced hyperinsulinemia was observed in MA mice on high fat diet. This suggests that MA mice, as compared to A and YA, are more prone to develop diet‐induced insulin resistance.

Our feeding trial demonstrates that the susceptibility to fatty liver development is established during pubertal maturation. High fat feeding for 9 weeks did not increase hepatic triglyceride content in A mice, whereas YA mice already showed the same degree of triglyceride accumulation as MA mice (Fig. 1A). Hepatic triglyceride content is strongly correlated with histochemical lipid droplet staining in hepatocytes as demonstrated in a previous study feeding the same diets (Ludwig et al., 2013). Our results expand published observations describing higher susceptibility to develop NAFLD in middle‐aged high fat‐fed mice (Sheedfar et al., 2014) and accelerated development toward steatohepatitis (Fontana et al., 2013).

A strong association of fatty liver development with mitochondrial function was identified characterized by the inverse relationship of age‐ and diet‐associated changes in mitochondrial mass and hepatic triglyceride content (Fig. 1A and B). We observed a decrease in mitochondrial mass in MA mice fed the control diet, which was in line with a previous report of reduced citrate synthase activity in liver of aging rats (Cahill et al., 2005). Reduced mitochondrial biogenesis is an accepted feature of aging (Lopez‐Otin et al., 2013). Intriguingly, mitochondrial mass was also lowered in YA mice fed the high fat diet, but not the control diet. This observation demonstrates that the dietary fat intake greatly prepones age‐associated changes in mitochondrial biogenesis (Fig. 1B, significant age × diet interaction). In addition, regarding bioenergetic function, the fatty acid oxidation capacity of liver mitochondria isolated from high fat‐fed mice was reduced, which was previously suggested to promote hepatic lipid accumulation (Vial et al., 2011; Garcia‐Ruiz et al., 2014). On the organ level, less mitochondria with reduced fatty acid oxidation capacity are likely to facilitate triglyceride accumulation in the high fat‐fed YA and MA mice. Interestingly, diet‐induced reduction in oxidation capacity was absent, when isolated mitochondria were fuelled with succinate, which indicates that TCA cycle function is not blunted due to high fat diet feeding, as observed previously (Satapati et al., 2012).

In a long‐term high fat feeding model deficiently assembled respiratory complexes have been identified (Garcia‐Ruiz et al., 2014), whereas our proteome analysis did not reveal systematic changes in mitochondrial proteins of the OXPHOS machinery, which could explain the observed changes on the bioenergetic level. However, we found reduced abundance of trifunctional protein in livers of high fat‐fed mice, in line with a previous report by others (Xiong et al., 2014). Trifunctional protein catalyzes several consecutive cycles of beta‐oxidation with high affinity for fatty acids of a chain length between 10 and 16 (Wanders et al., 1999), reflecting the main fatty acids in our high fat diet. Therefore, even a small reduction in the abundance of trifunctional protein could slow down mitochondrial fatty acid oxidation and limit the acetyl‐CoA pool. Additionally, some peroxisomal fatty acid oxidation enzymes were also reduced, which has been associated with hepatic fat accumulation in the past (Gambino et al., 2011; Knebel et al., 2015).

Taken together, we conclude that an age‐related reduction in mitochondrial mass potentially exacerbates the diet‐related reduction in mitochondrial function in high fat‐fed mice. Diminished capacity in various routes of fatty acid utilization further contributes to fatty liver development upon high fat feeding.

Our functional analysis did not reveal an influence of the dietary intervention on membrane potential and proton leak in isolated hepatic mitochondria in contrast to chronic metabolic disturbances in different mouse models of obesity (Franko et al., 2014). ROS production rates of liver mitochondria, however, were increased by high fat diet, independently of oxygen consumption or membrane potential. These findings further strengthen the overall hypothesis that elevated intake of dietary fats alters bioenergetic functions of liver mitochondria and results in mitochondrial‐derived oxidative stress (Begriche et al., 2013; Yu et al., 2014; Kakimoto and Kowaltowski, 2016). Elevated mitochondrial ROS production was previously reported in rodent studies, either directly detected in isolated liver mitochondria (Nadal‐Casellas et al., 2010; Ruiz‐Ramirez et al., 2011; Vial et al., 2011; Yu et al., 2014), or indirectly assessed by markers of hepatic oxidative stress (Raffaella et al., 2008; Ciapaite et al., 2011; Midha et al., 2012; Satapati et al., 2012; Yuzefovych et al., 2013). Of note, our experimental design using succinate to fuel mitochondria in the presence of Rotenone specifically detected ROS production at mitochondrial Complex III (cytochrome C reductase). Based on recent studies, an additional contribution of ROS generation by reverse electron flow at Complex I (NADH‐dehydrogenase) is also feasible, as demonstrated in skeletal muscle (Brand, in press) and liver (Vial et al., 2015).

To identify links between the molecular and functional levels, we conducted all assays using the same mitochondrial preparations. Due to limited yield of mitochondria from one liver, we measured ROS generation and oxygen production rates in individual mice, but pooled mitochondrial preparations of experimental groups for mass spectrometry‐based protein identification and quantification. Our mitochondrial preparations exhibited massive enrichment of annotated mitochondrial proteins (Fig. 3B), but at low abundance also contained proteins of endoplasmic reticulum, lysosomes, and peroxisomes. The number of mitochondrial proteins identified in isolates was on a competitive basis to other studies (Pagliarini et al., 2008; Deng et al., 2010; Guo et al., 2013; Nesteruk et al., 2014). Compared to the MitoCarta database (Pagliarini et al., 2008), the most complete compendium of mitochondrial proteins (Pagliarini and Rutter, 2013), our study identified an extended expression pattern of mitochondrial proteins in liver. The high purity of our mitochondrial preparations, as achieved by density‐gradient centrifugation, was demonstrated by a strong enrichment of mitochondrial proteins annotated to the outer membrane, intermembrane space, inner membrane, and mitochondrial matrix. This catalog will provide a valuable reference for the community.

In the pathway analysis, we identified the activation of NFE2L2‐regulated gene expression as a prominent response to the diet‐induced elevation of mitochondrial ROS production. Upregulated NFE2L2 targets involved in lipid‐hydroperoxide detoxification may be taken as an indicator for increased oxidative modifications of hepatic lipids in high fat‐fed mice. Intriguingly, our findings support the notion that NFE2L2 is tethered in a complex with its inhibitor to the outer mitochondrial membrane by PGAM5 (Xue et al., 2015). Although further validation of this interaction is required, the NFE2L2 protein complex may represent a favorable sensor for changes in the oxidative status in juxtaposition to the site of hydrogen peroxide release. Downregulation of PGAM5 was described to induce NFE2L2 translocation (Xue et al., 2015). Thus, our findings are compatible with ROS‐mediated activation of the mitochondria‐nucleus axis in early development of fatty liver.

Interestingly, SOD1 and catalase, both known targets of NFE2L2 (Zhang et al., 2015), were downregulated by high fat diet feeding. This unexpected finding may be explained by the observed upregulation of HSD11B1 in liver of high fat‐fed mice (Table 5). Hepatic glucocorticoid metabolism by this enzyme negatively regulates NFE2L2‐dependent gene expression (Kratschmar et al., 2012). In addition, oxidative stress adjusts the NFE2L2 response by enhancing the expression of other transcription factors competing for antioxidative response element binding (Kobayashi and Yamamoto, 2006). Beneficial effects of NFE2L2 activation on liver lipid metabolism are controversially discussed, however, the hypothesis of a balanced NFE2L2‐dependent gene expression putatively represents the most physiological state in livers of diet‐induced obesity, because neither constitutive activation, nor complete inactivation of NFE2L2 was shown to prevent diet‐induced obesity and NAFLD (Seo and Lee, 2013). A current hypothesis, mainly based on loss and gain of function studies in cell culture mouse models, suggests that NFE2L2 contains mitochondrial function and integrity (Dinkova‐Kostova and Abramov, 2015). Our data, reflecting the physiological range of regulation, do not provide further support for NFE2L2‐mediated enhancement of fatty acid oxidation or oxidative phosphorylation (Dinkova‐Kostova and Abramov, 2015). Conclusively, mitochondria‐derived oxidative stress could contribute to a balanced NFE2L2 mediated induction of a hepatic antioxidative response. Here, our study is limited to the effect of diet, because age‐dependent effects on NFE2L2‐related gene regulation were not assessed.

## Conclusions

By applying a cross‐sectional study design, we elucidated that the susceptibility to develop diet‐induced fatty liver increased with the age of mice, delineated by the acceleration of the age‐related decline in mitochondrial mass by feeding the high fat diet, and diet‐induced reduction in mitochondrial fatty acid oxidation. The parallel assessment of functional data and proteomic analysis identified NFE2L2 as a key regulator of the antioxidative response to high fat diet‐induced mitochondrial ROS production in liver. In future work the role of NFE2L2 should be further validated including age‐ and diet‐related effects. In general, the effects of aging on metabolism must be taken into account in the attempt to elucidate the etiology of NAFLD.

## Conflict of Interest

Authors have nothing to disclose.

## Data Files and Data Accessibility

Supplemental Table S1 displays all proteins regulated by high fat diet. Attributed biological functions are shown for selected proteins. In an extra register all proteins regulated by age are listed. In supplemental Table S2 all identified proteins are listed including the raw data as normalized intensities for all biological and technical replicates.

## Supporting information




**Table S1.** Regulated proteins by diet in isolated liver mitochondria. Compartments: M, mitochondrium; ER, endoplasmic reticulum; P, peroxisome; R, ribosome; L, lysosome; V, vesicle; Ct, cytosol; FC, fold change; HF, high fat; C, control.

**Table S2.** Normalized label‐free intensities of all identified proteins.
